# Improvement in Bending Strength of Silicon Nitride through Laser Peening

**DOI:** 10.3390/ma15010315

**Published:** 2022-01-02

**Authors:** Kazuya Saigusa, Joji Yamamoto, Koji Takahashi, Fumiaki Kumeno, Norihito Shibuya

**Affiliations:** 1 Graduate School of Engineering, Yokohama National University, Yokohama 240-8501, Japan; saigusa.kazuya.xiqzt@showadenko.com (K.S.); yamamoto-joji-gy@ynu.jp (J.Y.); 2 Faculty of Engineering, Yokohama National University, Yokohama 240-8501, Japan; 3 Sintokogio, Ltd., Nagoya 450-6424, Japan; f-kumeno@sinto.co.jp (F.K.); n-shibuya@sinto.co.jp (N.S.)

**Keywords:** laser peening, silicon nitride, compressive residual stress, bending strength

## Abstract

This study aimed to improve the bending strength and reliability of ceramics using laser peening (LP). In the experiment, LP without coating (LPwC) and with coating (LPC) were applied to silicon nitride (Si_3_N_4_) under various conditions. The surface roughness, residual stress, and bending strength were then measured for the non-LP, LPwC, and LPC specimens. The results show that the LPwC specimen had a greater surface roughness but introduced larger and deeper compressive residual stress when compared with the non-LP and LPC specimens. In addition, the bending strength of the LPwC specimen was higher and scatter in bending strength was less compared with the non-LP and LPC specimens. This may be attributed to the transition of the fracture initiation point from the surface to the interior of the LPwC specimen because of the compressive residual stress introduced near the surface. Thus, it was demonstrated that the application of LP is effective in improving the strength and reliability of ceramics.

## 1. Introduction

Ceramics are used in the manufacturing of sliding parts and high-temperature structural parts owing to their excellent heat, wear, and corrosion resistance when compared with metals. However, ceramics are more susceptible to defects than metals, which reduces their reliability and service life owing to the generation of small surface defects introduced during processing and use. It has previously been reported that shot peening (SP) introduces compressive residual stress and improves apparent fracture toughness [1,2,3]. Itoh et al. reported that SP improves the bending strength of partially stabilized zirconia (PSZ) by approximately 130 MPa [1]. Moon et al. showed that SP can be applied to silicon nitride (Si_3_N_4_) to introduce compressive residual stress and improve the apparent fracture toughness [2]. Tanaka et al. reported that fine particle peening and ultrasonic peening can be applied to Si_3_N_4_ to improve the apparent fracture toughness [3]. Furthermore, Pfeiffer et al. reported that the compressive residual stress introduced by SP increases the static and cyclic load capacities of alumina (Al_2_O_3_) and Si_3_N_4_ [4]. Takahashi et al. showed that SP can improve the contact strength of Si_3_N_4_ composites by at least nine times [5]. Iwanaka et al. showed that SP can improve the bending strength of PSZ by approximately 100 MPa [6]. Koike et al. clarified that the abrasion resistance can be improved by introducing compressive residual stress through SP [7], while Shukla et al. reported an improvement in the fracture toughness value of zirconia through fine particle peening [8].

However, owing to the physical contact with the shot material during SP, there is a possibility of surface peeling or chipping, which can considerably decrease material strength. In recent years, it has been reported that compressive residual stress can be introduced into ceramics without physical contact with the shot material through laser peening (LP). LP is a process in which a material placed in a transparent medium such as water or a water film is irradiated by a laser with a pulse width of a few nanoseconds to a few tens of nanoseconds to generate a high-pressure plasma, which is then used to peen the material surface using its impact force [9,10]. Akita et al. reported that LP can introduce compressive residual stress into Si_3_N_4_ [11]. Shukla et al. reported that LP improves the hardness and fracture toughness of Al_2_O_3_ [12] and Si_3_N_4_ [13]. Shukla et al. also reported that LP can introduce compressive residual stress and confirmed a considerable increase in dislocation density after LP in Al_2_O_3_ [14]. Wang et al. reported that LP can introduce a compressive residual stress of 900 MPa in Al_2_O_3_, which was retained after heat treatment [15]. Saigusa et al. reported on the optimum LP conditions for Si_3_N_4_/SiC composites [16]. However, in previous studies, change in bending strength of ceramics after LP application had not been investigated, except by Akita et al. [11]. Surface damage can be prevented by applying a coating called a sacrificial layer during LP construction and, in previous studies, laser peening with coating has been widely used for ceramics [12,13,14,15,16,17,18,19]. However, the changes in residual stress and bending strength with and without coating are also not clear. Moreover, the effects of LP on the scatter of bending strength are not clear.

Therefore, this study intended to clarify the effect of LP on the bending strength of ceramics. In the experiment, Si_3_N_4_ was subjected to LP, and the change in bending strength and its scatter were evaluated. Specimens with and without a coating were prepared for comparison, and their surface roughness, residual stress, and bending strength were measured, and the fracture surface after bending test was observed and discussed.

## 2. Experiments

### 2.1. Specimens and LP Conditions

**Figure 1:**
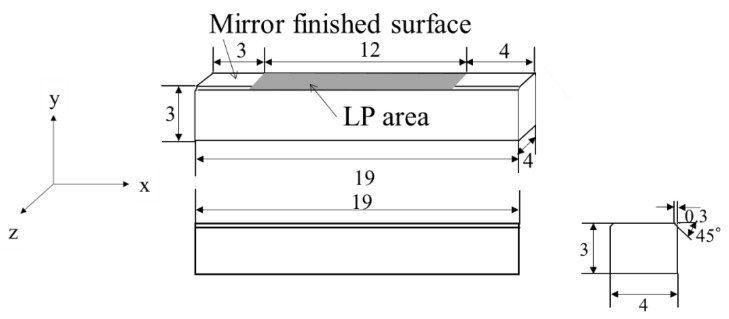
Shape and dimensions of bending test specimen (unit: mm).

The Si_3_N_4_ test specimen used was SN-1, manufactured by Japan Fine Ceramics Center (Nagoya, Japan) [20]. For the bending tests, specimens with dimensions of 3 mm × 4 mm × 19 mm were prepared, as shown in Figure 1. One side of the specimen is mirror-finished, which is referred to as the “non-LP” specimen in this paper. LP was then applied to this test specimen.

**Figure 2:**
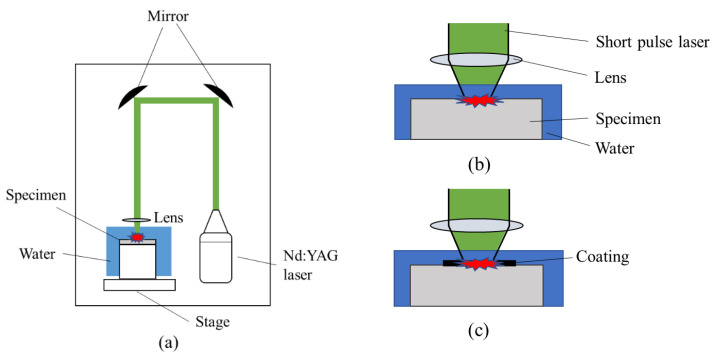
(**a**) Schematic of laser peening (LP) processes; (**b**) LP without coating (LPwC); (**c**) LP with coating (LPC).

Figure 2a shows the configuration diagram of the equipment used for LP processing, while Figure 2b,c shows the schematics of LP without coating (LPwC) and LP with coating (LPC). The surface of the LPC specimen was coated with black vinyl tape as a sacrificial layer. The pulse laser was reflected by a mirror and applied to a test specimen attached to a stage installed in a water tank through a condenser lens. LP processing was performed by fixing the test specimen to a jig for peening and moving the stage with the jig up, down, left, and right.

Table 1 shows the LP parameters used in the experiment. In the LP treatment, an Nd: YAG laser (SAGA, Thales, France ) with a wavelength of 532 nm was used, the repetition rate was 10 Hz, the spot diameter was 0.5 mm, and the number of passes indicating the number of laser paths was one or two. The power density, which indicates the light intensity per unit area, was set to 3 GW/cm^2^. These conditions were determined based on a previous study [16] and the results of our preliminary experiments.

**Figure 3:**
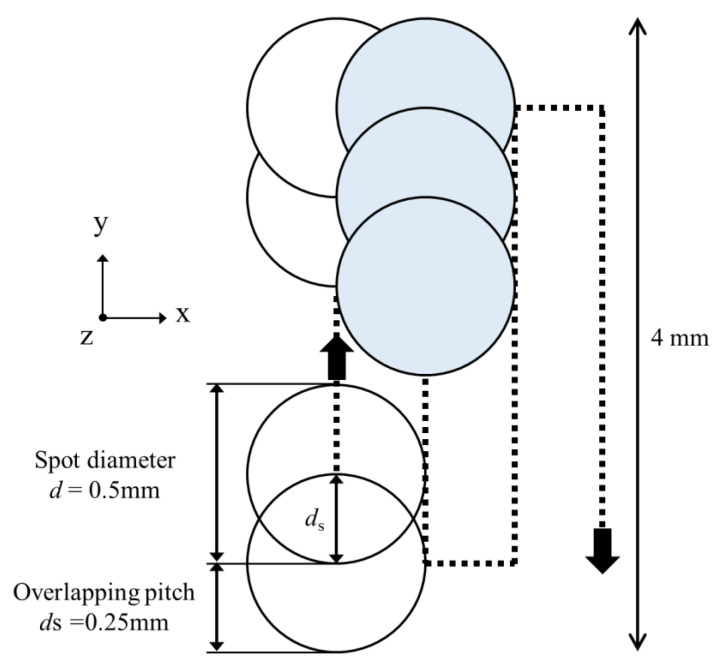
Peening pattern and laser track in the laser peened area.

LP treatment was performed in the area shown in Figure 1. The laser was irradiated while moving the stage on which the test specimen was placed. The laser was scanned such that the overlap ratio—the ratio between spot diameter and distance between centers—was 50% [15]. Figure 3 shows a schematic diagram of the spot when the overlap ratio is 50%. In this figure, the *x*-direction coincides with the longitudinal direction of the test specimen, and the *y*-direction coincides with the width direction of the test specimen. In this experiment, the laser was irradiated to reciprocate in the width direction of the test specimen. In the figure, the spot diameter (*d*) and the distance between the centers of the spots (overlapping pitch, *d*_s_) are shown. The value of *d*_s_ is the same in the *x*- and *y*-directions.

### 2.2. Experimental Procedure

**Figure 4:**
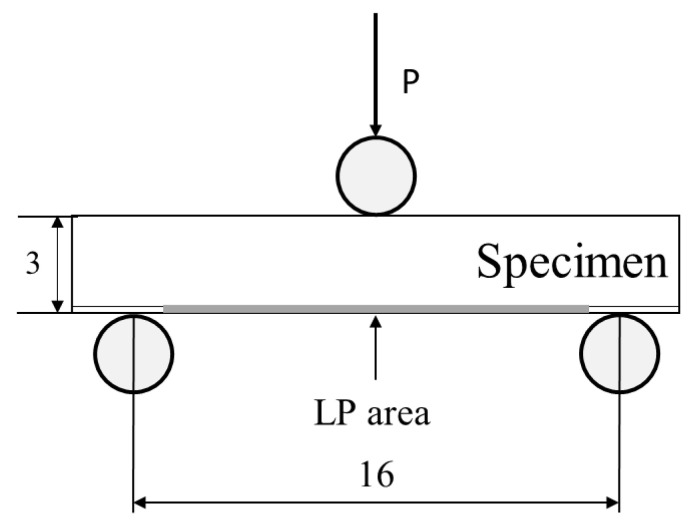
Schematic diagram of three-point bending test. (unit: mm).

The arithmetic mean roughness *Ra* in the longitudinal direction of the test specimen was measured using a stylus-type roughness meter (Kosaka Laboratory Ltd, Tokyo, Japan). The measurement was performed three times under each condition. The residual stress was measured using the cos *α* method under the conditions shown in Table 2. To investigate the residual stress introduced under each condition, the surface residual stress in the longitudinal direction of the test specimen was measured three times. In addition, to evaluate the residual stress distribution in the depth direction of the non-LP and LP specimens (LPwC-1 and LPC-1), the residual stress was measured by sequentially polishing in the depth direction. Diffraction of the (212) plane of Si_3_N_4_ by Cr-K*α* characteristic X-ray was used. The bending strength was measured by a three-point bending test at room temperature, as shown in Figure 4. The span length was 16 mm, and the crosshead speed was 0.5 mm/min. The fracture surface was observed via scanning electron microscope (KEYENCE, Osaka, Japan). The bending strength was evaluated using the Weibull distribution, based on the shape parameter *α* and the scale parameter *β*.

## 3. Results and Discussion

### 3.1. Surface Roughness of Specimens

**Figure 5:**
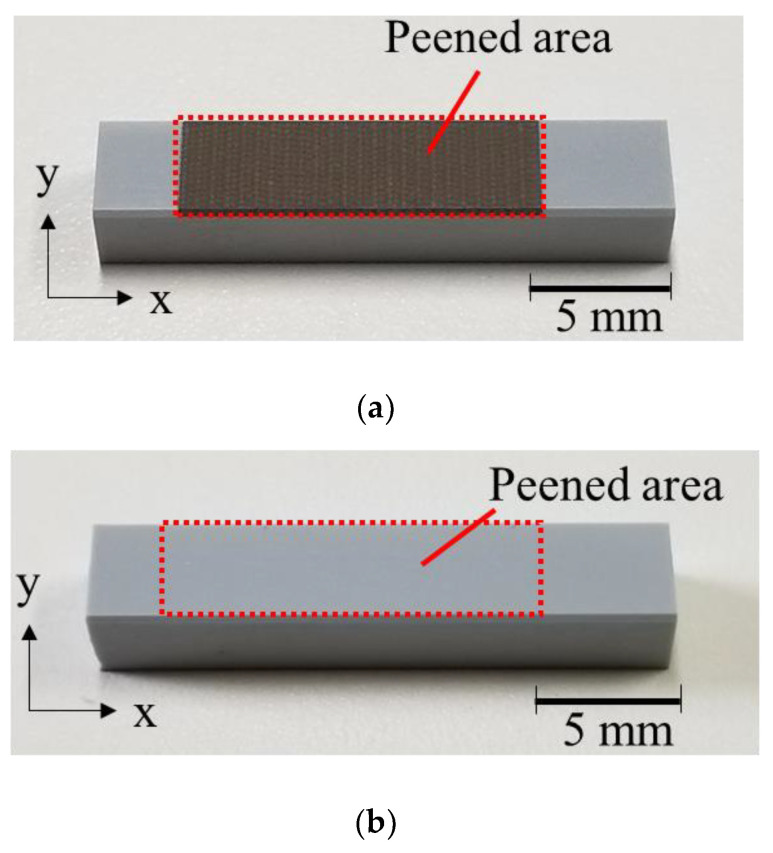
Photographs of LP specimens: (**a**) LPwC specimen (LPwC-1); (**b**) LPC specimen (LPC-1).

Figure 5a,b show macroscopic photographs of the LPwC and LPC specimens, respectively, where the central part of the test specimen is the peened area. On comparing (a) and (b), it can be observed that the LPwC specimen had circular spot marks on the surface of the test specimen that was irradiated with the laser. However, no changes due to LP were observed on the surface of the LPC specimen.

**Figure 6:**
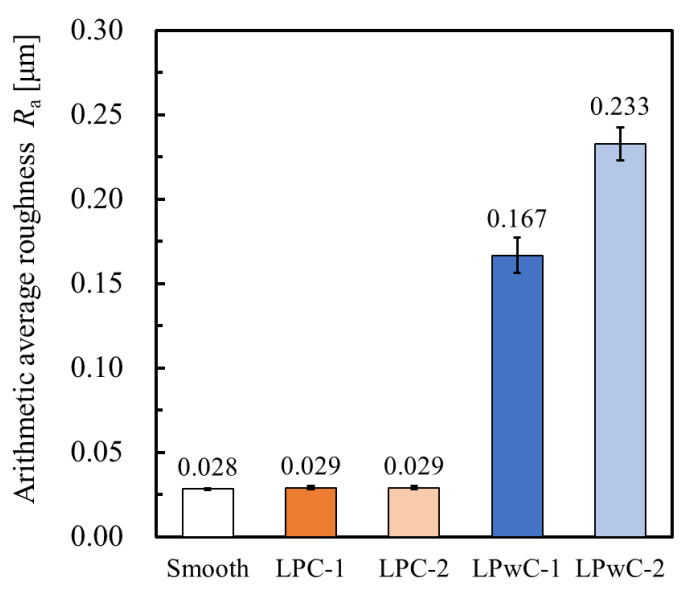
Comparison of surface roughness *Ra* of specimens.

Figure 6 shows the measurement results of surface roughness. The numbers indicate the average value of the measurement results of the three points. When the coating was applied, the surface roughness was equivalent to that of the non-LP specimen. This is because the laser ablation occurred above the coating in all LPC specimens, and the variation among specimens may have been small. In contrast, without coating, the surface roughness increased up to 0.233 μm. Since the roughness increases as the number of passes increases, it is considered that this is due to the duplication of laser ablation. The variation of the surface roughness in LPwC was larger than that of non-LP and LPC. The variation of surface roughness may be caused by the difference in the laser irradiation of each specimen due to the application of LP without coating.

**Figure 7:**
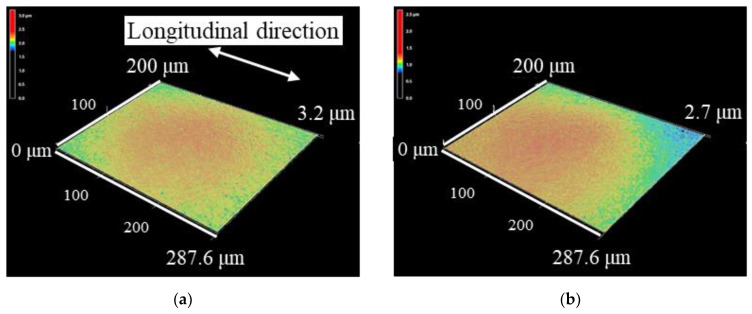

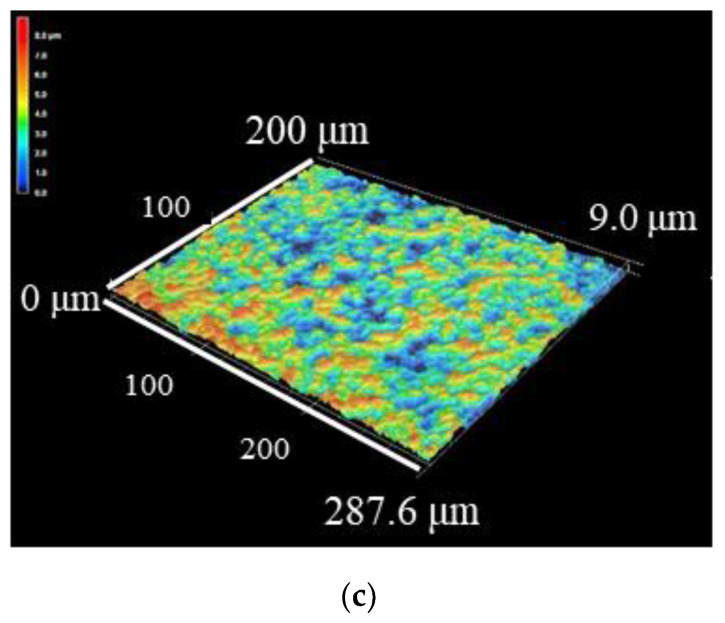
3D profiles of the surface of Si_3_N_4_: (**a**) Non-LP specimen; (**b**) LPC-1; (**c**) LPwC-1.

Figure 7 shows the 3D profiles of the non-LP and LP specimens in which the central part of the test specimen was observed using a laser microscope. Figure 7a–c show the observation results of the surface of the non-LP, LPC-1, and LPwC-1 specimens, respectively. From the appearance of (a) and (c), it can be confirmed that the surface roughness increased after LP. It can also be observed that the surface condition of the LPC specimen is similar to that of the non-LP specimen. This is because the surface of the LPC specimen in (b) is protected by the coating. The blue area in (c) for the LPwC material indicates that the surface of the material was damaged by ablation.

### 3.2. Residual Stress

**Figure 8:**
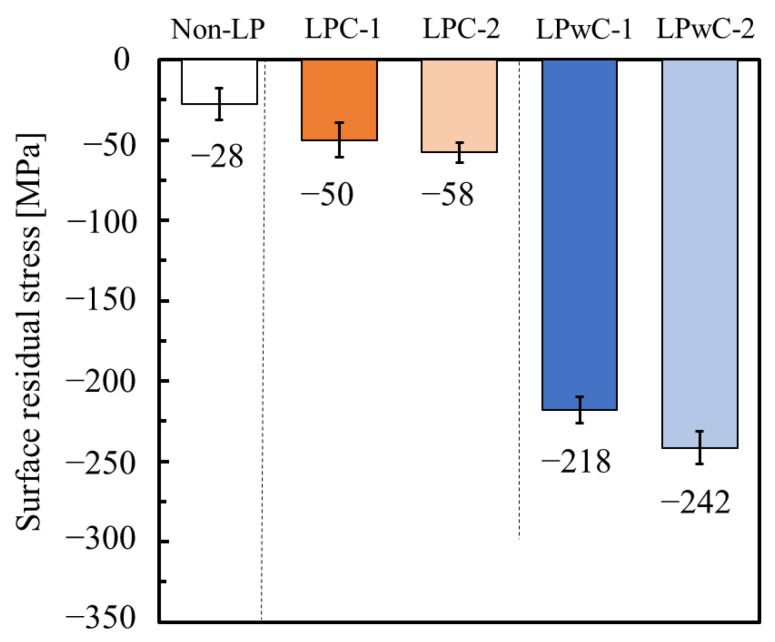
Comparison of the surface residual stress of specimens.

Figure 8 shows the residual stress on the surface of each specimen. Three points were measured near the center of the LP construction area, and the average value of the measurement results is shown by the number on the graph. The compressive residual stress of the non-LP specimen was as small as 28 MPa, which was introduced by machining the test specimen and polishing the surface. It was confirmed that compressive residual stress increased under all conditions in the test specimen after the application of LP. Thus, the residual stress was introduced on the surface using LP in the ceramic samples. Under the two conditions of LPC-1 and LPC-2, the values of compressive residual stress were approximately 50 MPa, which are larger than that of the non-LP specimen, but large residual stress was not introduced. In contrast, large compressive residual stress values of 218 and 242 MPa were introduced in LPwC-1 and LPwC-2, respectively. In addition, the residual stress increased with an increase in the number of passes. Wang et al. have reported that as the number of laser passes increased in alumina, the value of compressive residual stress increased, however, after reaching a peak value it decreased rapidly [21]. In our study, the coverage was not increased further, because increasing the coverage also increases the surface roughness (see Figure 6). When the coating was applied, the shock waves generated by the LP did not propagate far inside the specimen, and therefore the value of the compressive residual stress is smaller in LPC.

**Figure 9:**
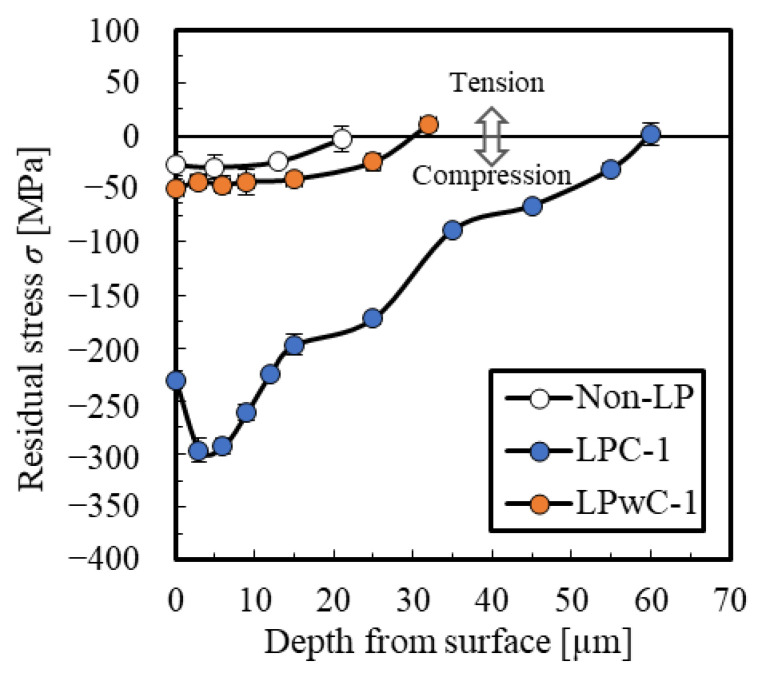
Residual stress distribution of each specimen.

Figure 9 shows the residual stress distribution in the depth direction of the non-LP specimen, LPC-1, and LPwC-1. The distance from the surface to zero residual stress point (the crossing point) in the non-LP specimen is approximately 20 µm. In LPC-1, the crossing point is approximately 30 µm. Although the value of the compressive residual stress increased slightly compared to the non-LP specimen, a large compressive residual stress was not introduced because the laser ablation occurred on the surface of the coating. In contrast, in LPwC-1, the crossing point was about 60 µm, and the compressive residual stress was introduced deeply. In addition, compressive residual stress of up to approximately 300 MPa was introduced. The compressive residual stress introduced into ceramics through SP is generally between 20 and 30 μm [3]. Therefore deep compressive residual stress was induced through LP in ceramics, similar to the case of metals

### 3.3. Bending Strength

**Figure 10:**
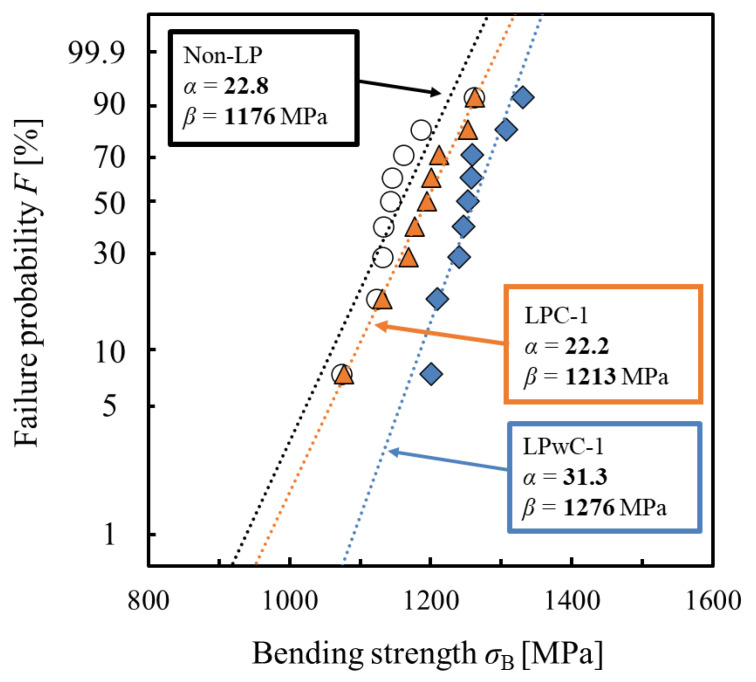
Weibull distribution of the non-LP and LP specimens.

To clarify the effect of LP on bending strength, bending tests were carried out on nine specimens (of non-LP specimen, LPwC-1, and LPC-1) each under three conditions. Based on the results, Weibull statistical analysis was performed. Figure 10 shows the two-parameter Weibull distribution of the bending strength of the non-LP specimens and LP specimens (LPwC-1 and LPC-1). The figure also shows the two parameters *α* and *β* obtained by linearly approximating each plot by the least-squares method. *α* is the shape parameter and indicates the degree of scatter of bending strength. *β* is a scale parameter and indicates $\sigma_{B}$ when the cumulative fracture probability *F* ($\sigma_{B}$) is 63%. The cumulative probability of failure is expressed by the following equation:

(1) $$F\left(\sigma_{B}\right)=1-\exp\left\{-{\left(\frac{\sigma_{B}}{\beta }\right)}^{\alpha }\right\}$$

The shape parameter α of LPwC-1 was 31.3, which is a significant increase from 22.8 for the non-LP specimen. In LPC-1, *α* was 22.2, which is almost the same value as *α* in the non-LP specimen. From these results, it can be said that the scatter in bending strength can be significantly reduced by applying LP without coating.

Furthermore, the scale parameter *β* was 1176 MPa for the non-LP specimen, while it was 1276 MPa for LPwC-1, indicating an improvement of 8.5%. Additionally, the scale parameter for LPC-1 was 1213 MPa, which is higher than that of the non-LP specimen. Although it increased by 3.1%, the improvement is still smaller than that observed for LPwC-1. From these results, it can be confirmed that LP can improve the bending strength as well as reliability by reducing the scatter of bending strength. In addition, a comparison between the LPC and LPwC specimens shows that LP without coating is more effective in achieving higher reliability and strength.

### 3.4. Fracture Surface Observation

**Figure 11:**
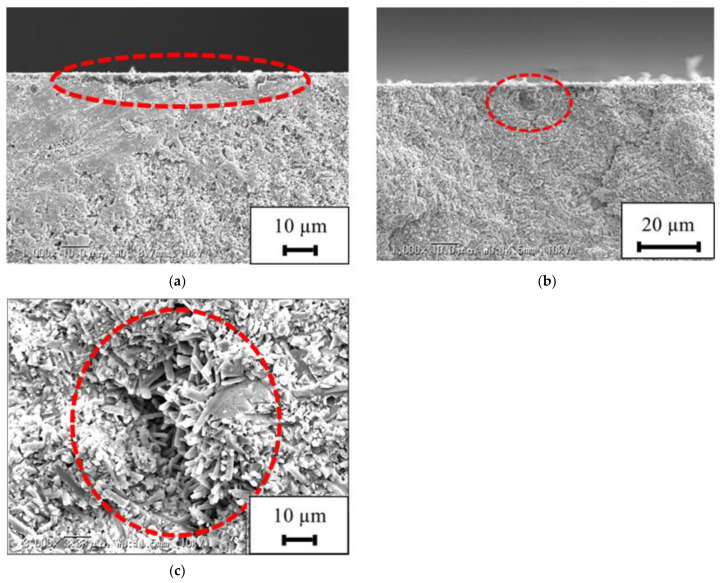
Fracture surface and fracture origin: (**a**) Non-LP specimen (*σ*_B_ = 1123 MPa; surface fracture origin); (**b**) LPC-1 specimen (*σ*_B_ = 1192 MPa; surface fracture origin); (**c**) LPwC-1 specimen (*σ*_B_ = 1201 MPa; inner fracture origin at depth of 38 µm).

Fracture surface observation was performed on the test specimen for which the Weibull distribution was obtained. The fracture surface of the specimens, non-LP, LPC-1, and LPwC-1, are shown in Figure 11a–c, respectively. On comparing Figure 11a,c, it can be observed that the fracture origin is located deeper in LPwC-1 than in the non-LP specimen. In LPC-1, the fracture origin is located on the surface similar to the non-LP specimen, as can be seen from Figure 11b.

**Figure 12:**
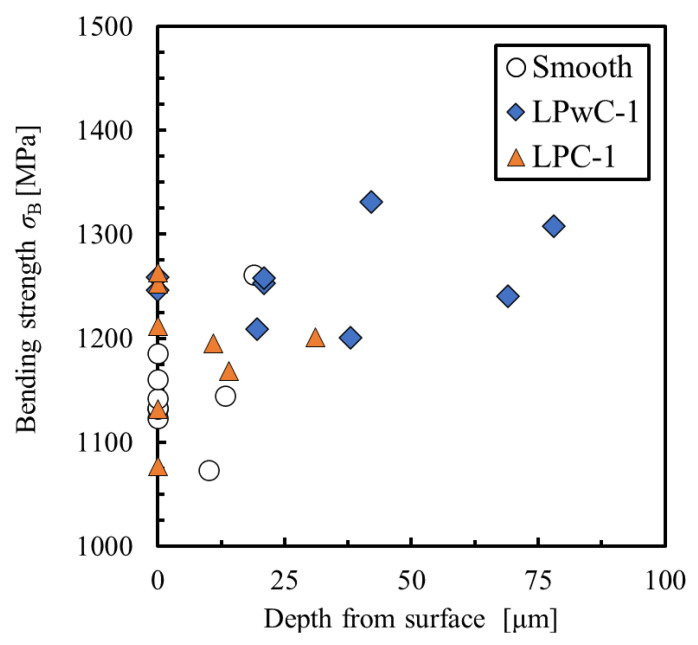
Relationship between bending strength and depth of fracture origin from surface of the specimen.

Figure 12 shows the relationship between the bending strength and depth from the surface of the fracture origin. In non-LP specimen, the surface fracture origin was 5 out of 9, while in LPwC-1, it was 2 out of 9. In LPC-1, it was 6 out of 9, and many of them were surface fracture origin. In addition, the maximum depth of the fracture origin of the non-LP specimen was approximately 25 μm, whereas the fracture origin of LPwC-1 was deeper than 60 μm. These results indicate that the compressive residual stress introduced by LP suppressed the crack initiation from the surface and, as a result, the fracture origin shifted to inside of the material in LPwC-1. In LPC-1, the maximum depth of the fracture origin was approximately 30 μm, which is similar to that of the non-LP specimen. This is probably due to the shallower depth of introduction of compressive residual stress in LPC-1 than in LPwC-1. Nevertheless, the bending strength of LPC-1 was improved compared to that of Non-LP. The reason for this is the effect of the compressive residual stress introduced by LP. Smyth et al. reported that the introduction of compressive residual stress into the aluminum alloy A2024-T351 by LP suppresses the growth of fatigue cracks and prolongs fatigue life [22]. In addition, Takahashi et al. reported that the introduction of compressive residual stress into aluminum alloy A7075-T651 by LP caused the fracture origin to transition to the interior, resulting in improved fatigue strength [23]. Thus, LP can suppress the crack growth and surface fracture in ceramics as well as in metals.

## 4. Conclusions

In this study, laser peening (LP) on various properties of silicon nitride (Si_3_N_4_) was evaluated to clarify its effect on the increase in strength and reliability of ceramics. The residual stress, surface roughness, and bending strength were measured through LP of Si_3_N_4_ under various conditions. To prevent ablation of the specimen surface by LP, a coated (LPC) and an uncoated specimen (LPwC) were prepared. The major results and findings are presented below.

(1) The results of residual stress and surface roughness measurements on LPwC and LPC specimens showed that large and deep compressive residual stress can be introduced when the coating is not applied, although the surface roughness increases.
(2) The bending strength of the specimens increased in the order of non-LP, LPC, and LPwC specimens. For LPwC, the scatter of bending strength improved compared with that of the non-LP specimen. However, for LPC, the scatter in bending strength was similar to that of the non-LP specimen.
(3) Fracture surface observations showed that a higher proportion of specimens in the LPwC specimen fractured due to internal defects than in the non-LP specimen. It is considered that the compressive residual stress introduced by the LP suppresses the crack growth from the surface, resulting in a more internal transition of the fracture origin. This transition of the fracture origin to the inside of the specimen is considered to be the reason for the improvement in bending strength in the LPwC specimen.
(4) The proportion of specimens that fractured from an internal origin in the LPC specimen was similar to that in the non-LP specimen, probably due to the shallower depth of introduction of compressive residual stress in the LPC specimen compared to the LPwC specimen. The slight increase in bending strength in LPC specimens can be attributed to the effect of the compressive residual stress introduced by LP.

These results showed that LP is an effective surface modification technique to improve the strength and reliability of ceramics.

## Figures and Tables

**Table 1:** LP parameters.

	LPC-1	LPC-2	LPwC-1	LPwC-2
Laser	Nd:YAG laser			
Pulse duration (ns)	6.2			
Repetition rate (Hz)	10			
Spot diameter (mm)	0.5			
Pulse energy (mJ)	37			
Irradiation density (pulse/mm^2^)	16			
Power density (GW/cm^2^)	3			
Overlap ratio (%)	50			
Overlapping pitch (mm)	0.25			
Path	1	2	1	2
Coating	With	With	Without	Without

**Table 2:** Residual stress measurement condition.

Measuring Method	Cos αMethod
Characteristic X-ray	Cr-Kα
Diffraction angle [deg]	131.614
Diffraction plane	Si_3_N_4_ (212)
X-ray irradiation time [s]	60
Incident angle [deg]	29
Tube voltage [kV]	30.0
Tube current [mA]	1.0

## Data Availability

Not applicable.
